# Using Simulation to Interpret a Discrete Time Survival Model in a Complex Biological System: Fertility and Lameness in Dairy Cows

**DOI:** 10.1371/journal.pone.0103426

**Published:** 2014-08-07

**Authors:** Christopher D. Hudson, Jonathan N. Huxley, Martin J. Green

**Affiliations:** School of Veterinary Medicine and Science, University of Nottingham, Sutton Bonington Campus, Sutton Bonington, Leicestershire, United Kingdom; University of Bonn, Germany

## Abstract

The ever-growing volume of data routinely collected and stored in everyday life presents researchers with a number of opportunities to gain insight and make predictions. This study aimed to demonstrate the usefulness in a specific clinical context of a simulation-based technique called probabilistic sensitivity analysis (PSA) in interpreting the results of a discrete time survival model based on a large dataset of routinely collected dairy herd management data. Data from 12,515 dairy cows (from 39 herds) were used to construct a multilevel discrete time survival model in which the outcome was the probability of a cow becoming pregnant during a given two day period of risk, and presence or absence of a recorded lameness event during various time frames relative to the risk period amongst the potential explanatory variables. A separate simulation model was then constructed to evaluate the wider clinical implications of the model results (i.e. the potential for a herd’s incidence rate of lameness to influence its overall reproductive performance) using PSA. Although the discrete time survival analysis revealed some relatively large associations between lameness events and risk of pregnancy (for example, occurrence of a lameness case within 14 days of a risk period was associated with a 25% reduction in the risk of the cow becoming pregnant during that risk period), PSA revealed that, when viewed in the context of a realistic clinical situation, a herd’s lameness incidence rate is highly unlikely to influence its overall reproductive performance to a meaningful extent in the vast majority of situations. Construction of a simulation model within a PSA framework proved to be a very useful additional step to aid contextualisation of the results from a discrete time survival model, especially where the research is designed to guide on-farm management decisions at population (i.e. herd) rather than individual level.

## Introduction

The ever-growing volume of data routinely collected and stored in everyday life presents researchers with a number of opportunities to gain insight and make predictions. Routine collection of data with potential research application is now very widespread, and is facilitating research using much larger sample sizes than in the past. One example of this is in agriculture, where widespread adoption of computerised recording systems has largely been driven by the need to manage larger enterprises and maximise efficiency, but is also creating an invaluable resource for researchers. A wide variety of traditional and new techniques have been applied to analysis of the large, retrospective datasets generated in this way, but in some cases more sophisticated and robust analytical techniques can yield results which are harder for the end user of the research to interpret and understand.

This study focuses on the relationship between a time-to-event outcome (in this case, the time between parturition and subsequent conception in a dairy cow) and a disease event (in this case lameness). Techniques for analysis of such data have evolved over the years, and this specific field has seen publications evaluating this relationship in a univariate way [1] using Kaplan-Meier survival analysis, and in a multivariate framework, using various modifications of the Cox proportional hazards model [2]–[4]. However, accounting appropriately for time-dependent variables (for example, accounting for the possibility that a case of lameness may affect probability of conception within a specific frame of time around the case) using such approaches can be challenging, and model assumptions can be difficult to satisfy and are not always tested [5].

Another approach is discrete time survival analysis [6], [7], where the dataset is amplified into smaller units of time for each individual animal and logistic regression is used to predict the probability of the outcome of interest at each time-point. This method is substantially more flexible, and more easily incorporates statistical advances such as multilevel regression using random effects to account for hierarchical clustering within data [7], [8] (for example, of cows within herds), and Markov chain Monte Carlo sampling for parameter estimation within a Bayesian framework [9]. However, results from this type of analysis can be difficult to interpret, especially at the population level. For example, such analysis may yield an estimated odds ratio for the association between a lameness event and the probability of conception occurring during a given period of time, but there is no intuitive way to interpret the likely importance of this at the population level. In this context, on-farm interpretation is very important, because decision makers (e.g. a dairy herd’s manager or veterinary clinician) need to be able to estimate the potential improvement in a herd’s reproductive performance that could result from a reduction in lameness in order to conduct a cost benefit analysis for intervention. Simulation based approaches can be used to help address this issue, allowing the researcher to evaluate relationships between inputs and outputs of a given system across plausible scenarios. One such technique is known as probabilistic sensitivity analysis (PSA), and is commonly employed in health economic evaluations [10], [11] to explore which inputs to a complex model have the most capacity to perturb the output. Such simulation models can also be used as the basis for decision support tools, an application common in the financial sector [12].

Good reproductive performance is essential for efficiency in dairy production [13], which in turn is increasingly important in the context of a global increase in demand and downward pressure on resource use [14]. A wide range of factors are known to affect dairy cow fertility, including incidence of clinical disease. Lameness is one of the most common endemic diseases in the modern dairy herd, with reported prevalence in the UK at over 35% [15], and has previously been associated with depressed reproductive performance in affected cows compared to unaffected controls [2], [4], [16], [17]. However, a very high proportion of previous studies have been carried out using either a single herd or a small number of herds, and those deriving data from wider populations have often failed to detect an association [18], [19], as did the most recent study in UK dairy cows [1]. Alongside this, a very wide variety of other factors are known to affect cow fertility. Therefore the clinician wishing to improve a herd’s reproductive performance needs to interpret this research evidence in the context of the other influences on fertility when deciding how much weight should be given to control of lameness to improve reproduction.

In this study, the association between clinical lameness events and reproductive performance was evaluated using routinely collected management data from a group of dairy herds. The aim of the study was to explore the usefulness of simulation-based techniques as an aid to interpret the clinical significance of a discrete time survival model evaluating association between disease events and reproductive performance at herd level.

## Materials and Methods

### Data Collection and Restructuring

Routinely recorded farm management data were collected from 39 dairy herds across England and Wales. These were the subset of herds described in an earlier study [20] which demonstrated consistent recording of clinical lameness events (i.e. treatment of lame cows). Data collection, quality auditing and study inclusion criteria are described in detail by Hudson et al. [20]. Herds were not excluded on the basis of breed: 38 were mainly Holstein or Holstein–Friesian and one predominantly Guernsey. Detail regarding each event (for example, which limb was affected and the diagnosis made) was not evaluated in this study: all clinical lameness events were treated as equal. Where two lameness events were recorded for the same cow within 7 days, the second was removed (since both treatment records would have been likely to reflect the same disease event). Table 1 shows descriptive information for these herds.

**Table 1:** Summary statistics of basic herd information for 39 dairy herds with good fertility and lameness records.

			Percentiles			
	Mean	Minimum	25%	50%	75%	Maximum
Herd size	243	88	153	202	292	669
Cull rate (%/year)	22	13	20	22	25	31
305 day adjusted milk yield (litres)	8329	4776	7366	8266	9566	11008
Incidence rate of clinical lameness (cases/cow-year)	0.40	0.10	0.22	0.30	0.41	1.88

Data were restructured into a format where each unit (line) of data was a two-day period during each lactation between 20 and 220 days after parturition (days in milk, DIM) where the cow was “at risk” of becoming pregnant (lactations were censored after culling, death, sale or conception occurred). For each of these two-day risk periods, a binary variable was used to represent whether the cow became pregnant during the risk period. Clinical lameness records were used to determine whether a case of lameness was recorded at a variety of different time-frames relative to each risk period (see Table 2). Additional variables at both lactation level (e.g. parity of cow, lactation 305-day adjusted milk yield) and risk period level (e.g. DIM at beginning of risk period, month and year of risk period) were calculated for each risk period (Table 2). Where necessary, categorical variables were recoded to avoid categories containing small numbers of risk periods/lactations (e.g. animals of parity 5 or above were grouped as a single category). This generated a dataset consisting of 1,247,677 risk periods from 21,913 lactations in 12,515 cows from 39 herds. Initial data collation and restructuring was carried out using Microsoft Access 2010 (Microsoft Corp.), with further restructuring and variable calculation carried out using R v2.14 [21].

**Table 2:** Potential explanatory variables calculated for each risk period in a study investigating the association between lameness and fertility in 39 dairy herds.

Variable	Level	Variable type
Parity (lactation number)	Lactation	Categorical (>4 recoded as single group)
305-day lactation milk yield	Lactation	Continuous
Year in which lactation began	Lactation	Categorical (<2003 recoded as single group)
DIM at start of risk period	Risk period	Continuous
Season of risk period	Risk period	Categorical (Jan-Mar, Apr-Jun, Jul-Sep, Oct-Dec)
Lame 71–100 d before risk period	Risk period	Binary (lameness case recorded or not)
Lame 43–70 d before risk period	Risk period	Binary (lameness case recorded or not)
Lame 15–42 d before risk period	Risk period	Binary (lameness case recorded or not)
Lame within 14 d of risk period	Risk period	Binary (lameness case recorded or not)
Lame 15–42 d after risk period	Risk period	Binary (lameness case recorded or not)
Lame 43–70 d after risk period	Risk period	Binary (lameness case recorded or not)
Lame 71–100 d after risk period	Risk period	Binary (lameness case recorded or not)

### Discrete-time survival analysis

A multilevel discrete-time survival model [22] was constructed to evaluate the association between the probability of a cow becoming pregnant during a two-day risk period (the outcome) and the potential explanatory variables described in Table 2. A three-level hierarchical structure (with risk periods nested within cows nested within herds) was used to account for correlations between risk periods from the same cow and cows from the same herd.

The model took the standard form:
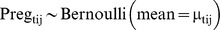





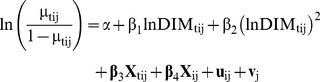
(1)




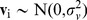
(2)




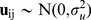
(3)where t represents a two-day risk period and i and j the i^th^ cow in the j^th^ herd; µ_tij_ the fitted probability of Preg_tij_ (the outcome of the i^th^ cow in the j^th^ herd becoming pregnant during risk period t); lnDIM_tij_ the natural logarithm of DIM at the beginning of risk period t; α the regression intercept; β_1_ and β_2_ the coefficients for the terms representing days in milk; **X**_tij_ the vector of risk period level covariates and **β**_3_ the corresponding vector of coefficients; **X**_ij_ the vector of cow-level covariates and **β**_4_ the corresponding vector of coefficients; u_ij_ the random effect to reflect variation between individual cows (e.g. due to genetic variation) and v_j_ the random effect representing variation between herds (e.g. due to nutritional management or environmental conditions of the herd), with 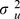 and 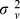 the variances of the normal distributions of the respective random effects terms.

Model building and final parameter estimation was carried out using MLwiN v2.20 [23]. Model building and selection used the approach described in Hudson et al. [20], with Markov chain Monte Carlo (MCMC) sampling used for final parameter estimation [9] and retention in the model of variables where the 95% area of highest posterior density (HPD) for the variable’s coefficient did not cover zero. Biologically plausible first order interaction terms were tested, and retained in the model only if their inclusion made a substantial difference to parameter estimates for coefficients of the main effects. Inclusion of herd-level random effects (slope variation) for the lameness-related model terms was also tested, to account for the possibility that the association between lameness and reproductive performance could vary between herds. These were again retained in the model only if they altered parameter estimates for main effects by more than 1%, or if between-herd variation was large relative to mean effect size (such that the variance of the herd-level random effect for the variable was more than 20% of the mean/overall effect).

Model sensitivity analysis revealed that the parameters of interest were not sensitive to choices made during data restructuring and model building (e.g. choice of risk period duration, choice of function to represent DIM or selection of timeframes for lameness events). Simulation-based posterior predictions were used to evaluate model fit as described in Hudson et al. [20], by subsetting the data in a variety of ways, using the model to predict probability of pregnancy for each risk period in the subset and checking that the observed proportion of risk periods where pregnancy occurred lay within the 95% coverage interval of the predicted risk. Model results were illustrated as relative risks using a similar prediction-based approach [20]. Posterior predictions were carried out in R v2.14, using MCMC chains exported from MLwiN.

### Probabilistic sensitivity analysis

In order to explore the relationship between herd reproductive performance and the incidence rate of lameness at herd level, a simulation model was developed. The aim of this part of the study was to evaluate the results of the discrete time survival analysis in a wider context to assess its potential usefulness to inform clinical on-farm management decisions.

#### Simulation model structure and process

**Figure 1:**
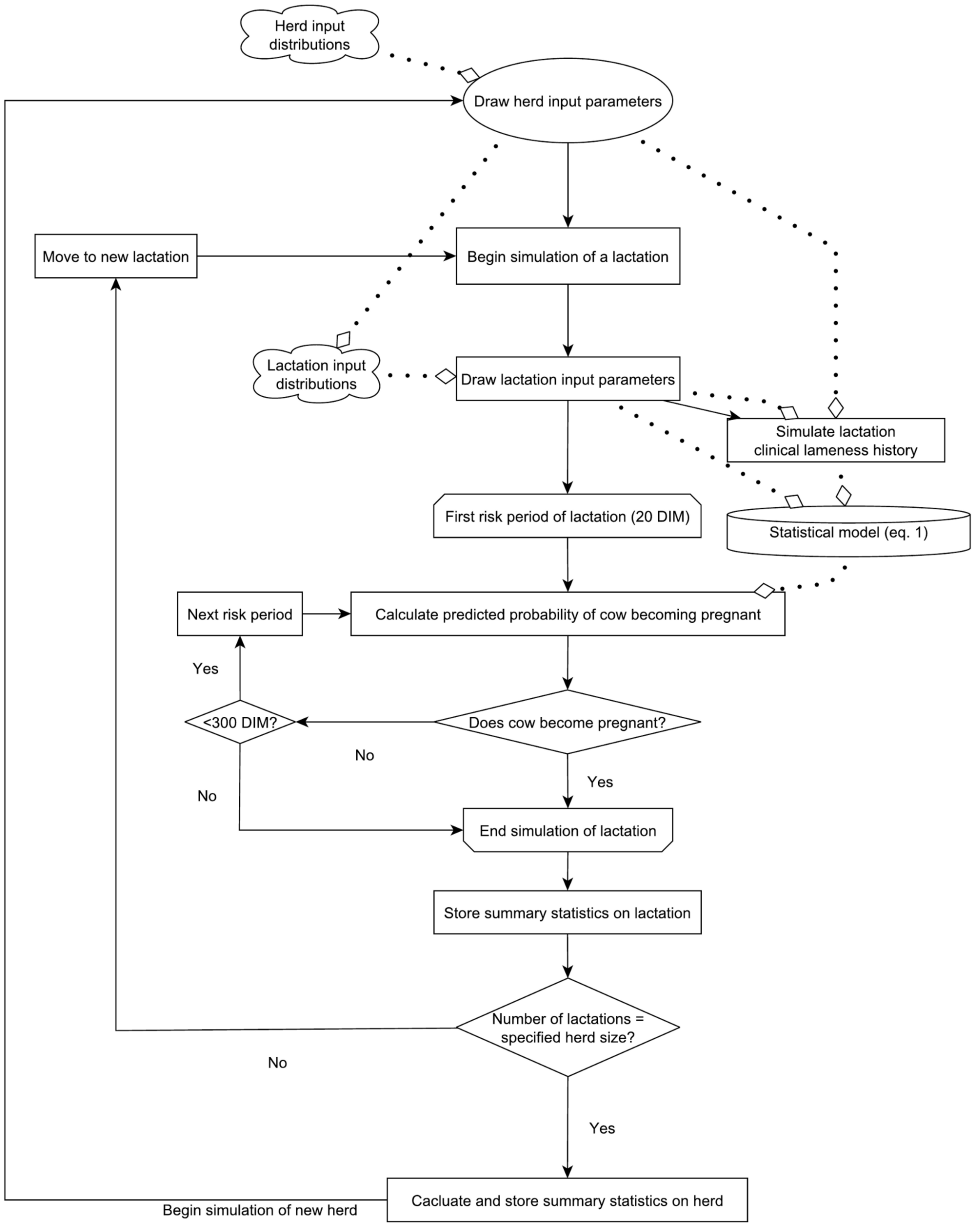
Structure of the simulation model used for probabilistic sensitivity analysis. Solid black lines indicate process flow, dotted lines indicate that information from the source of the line is used in the step of the process to which the line leads (denoted by a diamond).

The outline structure of the simulation model is shown in Figure 1. The model was constructed in Microsoft Excel 2010 (Microsoft Corp.), using Visual Basic for Applications (Microsoft Corp.) for process control. The explanatory variables in the final discrete-time survival model became input parameters for the herd-level simulation model, which was used to simulate 50,000 herds of 200 lactations each. Simulating a herd first involved drawing the herd-level input parameters (e.g. the herd’s mean 305-day adjusted milk yield and incidence rate of clinical lameness) from the distributions shown in Table 3.

**Table 3:** Input parameters used for each level of simulation in a study investigating the association between lameness and fertility.

Input variable	Level	Input distribution
Submission rate	Herd	Uniform, range 10–80%
Pregnancy rate	Herd	Uniform, range 10–60%
Herd average 305 d milk yield	Herd	Uniform, range 3000–12,500 litres
Proportion of herd in first lactation	Herd	Uniform, range 10–40%
Incidence rate of lameness	Herd	Uniform, range 0.1–1.5 cases/cow-year
Cost per extra empty day	Herd	Uniform, range £1.20–£4.20
Cost per failure to conceive cull	Herd	Uniform, range £550–£1750
Parity/lactation number	Lactation	Discrete, based on proportion of herd in first lactation
305 d lactation milk yield	Lactation	Beta, centred on herd average with standard deviation of 1,500 litres; adjusted for parity
Days in milk	Risk period	As described in text
Lame 43–70 d before risk period	Risk period	Binary, as described in text
Lame within 14 d of risk period	Risk period	Binary, as described in text
Lame 43–70 d after risk period	Risk period	Binary, as described in text
Lame 71–100 d after risk period	Risk period	Binary, as described in text

**Figure 2:**
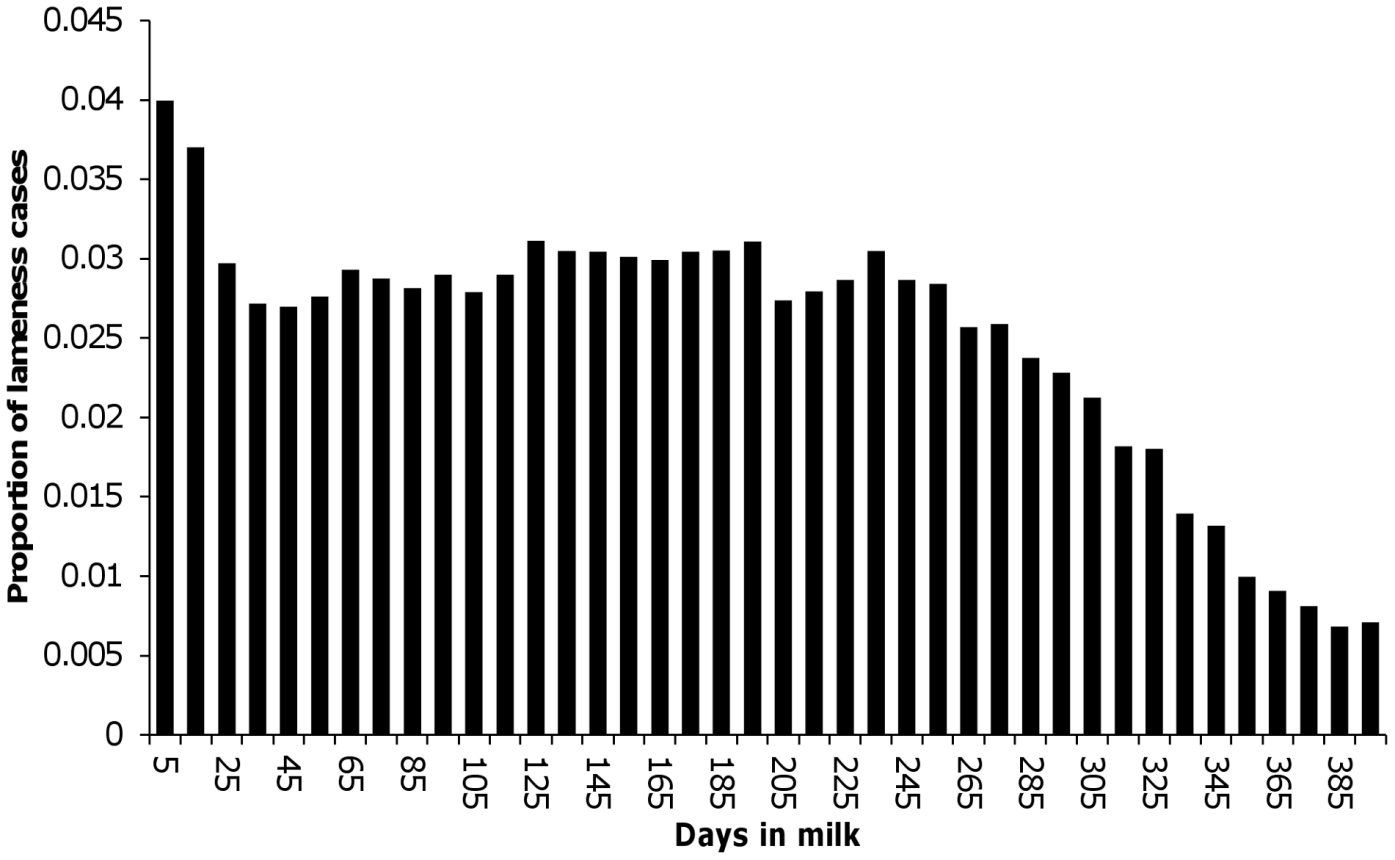
Distribution of lameness cases observed by days in milk.

Simulation of the first cow-lactation in the herd was then commenced by drawing the lactation-level inputs (e.g. the parity of the cow) from the relevant distributions and simulating a clinical lameness history for the lactation. The latter was accomplished by using the distribution of DIM of all clinical lameness events from the original dataset (Figure 2) to assign a probability that a lameness event would occur at each two-day risk period through the lactation in a herd with a given overall lameness incidence rate. The discrete-time survival model described in the previous section was used to calculate the predicted probability of pregnancy occurring during each two-day risk period given the input parameters for that herd, lactation and risk period. This probability was adjusted to account for the herd’s overall (“background”) level of submission rate and pregnancy rate (i.e. the variation in these parameters not explained by lameness, milk yield or other model inputs). These are measures of specific aspects of a dairy herd’s reproductive performance, submission rate being the proportion of eligible cows inseminated every 21 days (the normal length of the oestrous cycle) and pregnancy rate being the proportion of inseminations leading to a pregnancy. Both of these “background” herd fertility characteristics were represented as herd-level input parameters with a separate value for each simulated herd drawn from the relevant distribution.

A binary outcome to represent whether or not the cow became pregnant during the risk period was drawn from a binomial distribution based on this calculated probability. Repeated risk periods were simulated for each cow, until she either became pregnant or reached 300 DIM (a point at which farmers would commonly elect to remove cows from the herd if not pregnant), at which time simulation of the next lactation was begun. When 200 lactations had been simulated, the herd was considered complete. The mean number of DIM at pregnancy and the proportion of lactations ending without a pregnancy in each herd were stored along with the herd-level input parameters before beginning simulation of the next herd.

#### Simulation model inputs

Uniform input distributions were specified for all herd-level inputs, so that every potential combination of herd-level inputs was equally likely to be selected at each iteration of the simulation. The ranges for these distributions were selected based on the authors’ clinical experience, such that they would be expected to cover the vast majority of realistic possibilities in UK dairy herds (Table 3). This was not considered to represent the true joint distributions of these parameters across herds: the objective was not to speculate on which situations might occur most commonly, but to evaluate the potential impact of all different lameness incidence rates across as wide a variety of herd scenarios as possible. Some of the lactation-level inputs were drawn from non-uniform distributions so that the architecture of each simulated herd was realistic (so, for example, the milk yield for a lactation was drawn from a beta distribution parameterised such that a cow was likely to draw a lactation yield close to the herd average, and there was a smaller chance of drawing a yield much further from the average), as described in Table 3.

#### Simulation model outputs and analysis

A single herd-level outcome was devised to represent reproductive performance for each simulated herd (to allow evaluation of associations between this and the various input parameters). The mean number of DIM at pregnancy and the proportion of cows reaching 300 DIM without conceiving were combined using a modification of the method of Esslemont et al. [24] to produce a “modified FERTEX” (mFX) score. This involved comparing each value to a pre-set target (set at 60 days for the herd’s mean DIM at pregnancy and zero for proportion of cows in the herd reaching 300 DIM without conceiving), and applying a unit cost to the difference from target for each. The sum of these two costs on a per-cow basis for each simulated herd gave that herd’s mFX score. Since the cost of a culled cow and an additional empty day are widely acknowledged to vary from herd to herd, these were considered as herd-level inputs, and each drawn randomly for each herd from the distributions described in Table 3. The mFX score for each simulated herd was therefore a cost-based single measure of overall fertility performance (so that higher performing herds had lower mFX scores and vice versa).

Results from the simulations were analysed initially by illustrating associations between herd-level input parameters and mFX scores graphically using high-density scatterplots. Spearman rank correlation coefficients were calculated for the association between each herd-level input and mFX score (a non-parametric measure of correlation was selected as the mFX scores were positively skewed). Multiple regression (with the natural logarithm of mFX score as the outcome) was used to partition variance in mFX score between the various herd-level inputs. The resulting regression model was also used to predict the effect on mFX score of increasing each individual input in turn from the middle of its input distribution to the upper quartile so that results could be displayed graphically as a tornado plot (a standard approach for presentation of PSA results).

## Results

There were a total of 16,706 pregnancies from the 1,247,677 risk periods in the dataset, so that 1.34% of risk periods resulted in a pregnancy (corresponding to around 14% of cows becoming pregnant during each 21 day oestrous cycle). Of the 22,319 lactations in the dataset, 4,360 involved at least one case of lameness (corresponding to a lactational first case incidence rate of 19.5%).

### Discrete-time survival analysis

**Figure 3:**
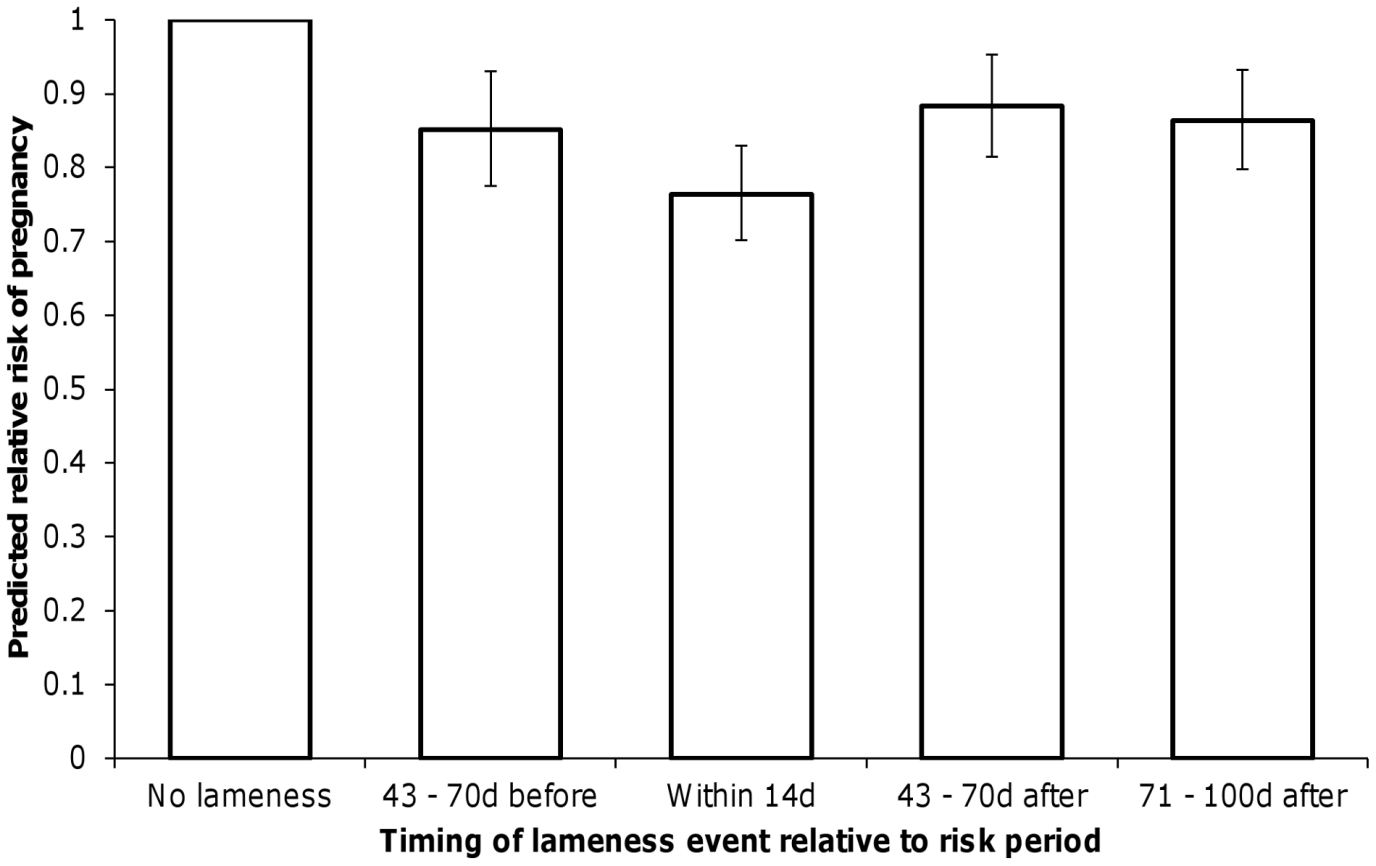
Association between predicted relative risk of pregnancy at a given risk period and clinical lameness. Error bars represent the 95% credible interval for each predicted relative risk.

**Figure 4:**
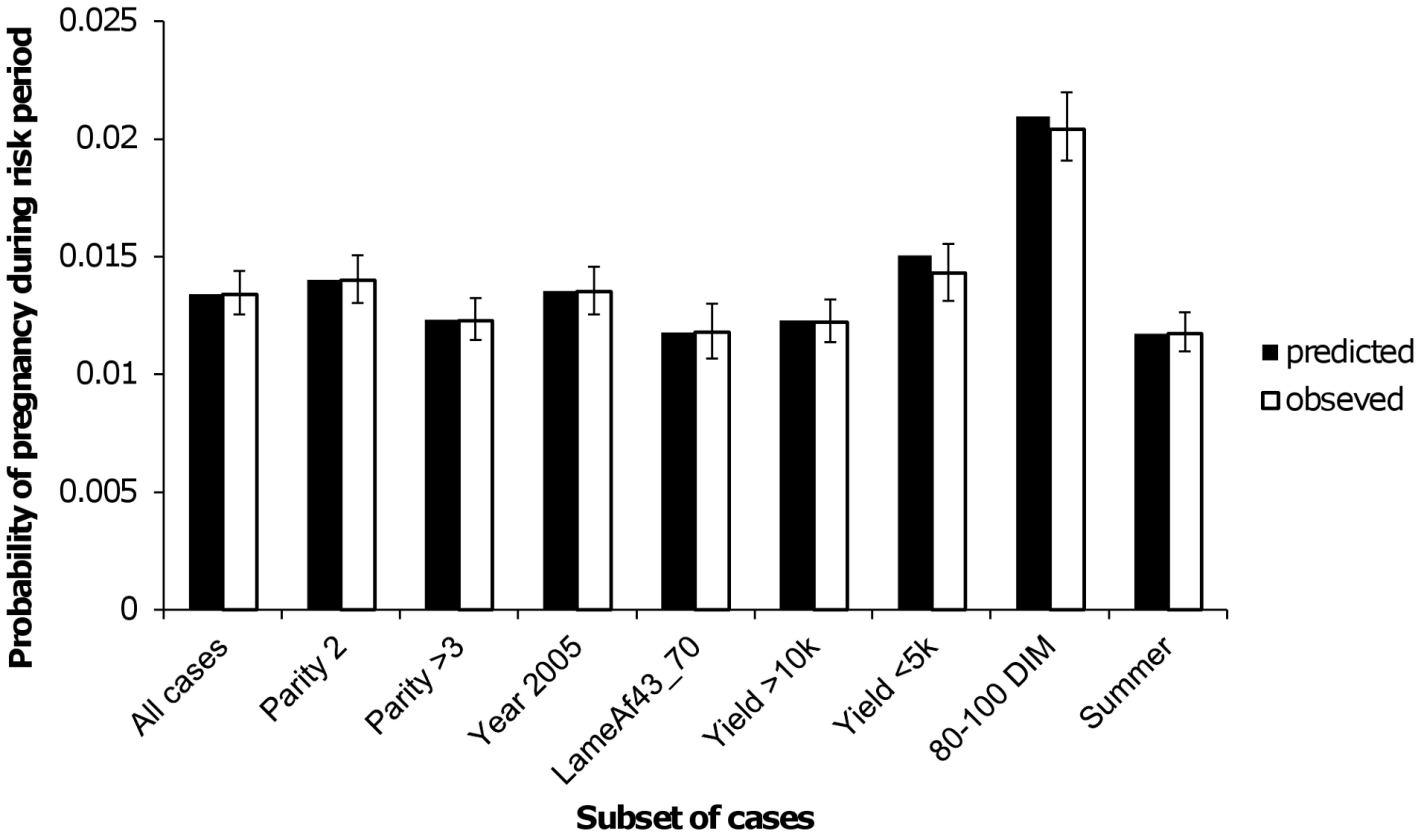
Predicted and observed risk of pregnancy across various categories. Predicted absolute risk of pregnancy (black bars) at risk periods in various categories (x-axis) compared to the observed proportion of risk periods in that category where a pregnancy occurred (white bars). Error bars represent the 95% credible interval for each predicted relative risk.

Table 4 shows the parameter estimates for the regression model derived to predict the probability of pregnancy resulting during a two-day risk period. The predictor variables not directly associated with lameness showed very similar associations to those seen by Hudson et al. [20], with probability of pregnancy peaking at around 110 DIM, decreasing with increasing 305-day adjusted milk yield and lower predicted probabilities of pregnancy for cows in higher parities and during the months April to September. Clinical lameness events during four different time frames relative to the two-day risk period showed associations with the probability of pregnancy during the risk period. The largest association was seen when a lameness event was recorded within 14 days of the risk period, when the odds of pregnancy were reduced by almost 25% (odds ratio [OR] 0.76, area of 95% highest posterior density [HPD] 0.69–0.84). Lameness events recorded 43 to 70 days before, 43 to 70 days after and 71 to 100 days after a risk period were all associated with a reduction in the odds of pregnancy during the risk period of around 15% (ORs 0.85, 0.88 and 0.86 respectively; areas of 95% HPD 0.76–0.95, 0.80–0.98 and 0.79–0.95 respectively). These associations are represented as posterior predicted relative risks in Figure 3. Predicted risks were also used to demonstrate that model fit was good. For each subset of data tested, the observed proportion of risk periods where pregnancy occurred fell within the 95% area of HPD of predicted risk for that subset (Figure 4).

**Table 4:** Parameter estimates for discrete time survival model with pregnancy during a two-day risk period as the outcome, in a study investigating the association between lameness and fertility in 39 dairy herds.

Model term	n	coefficient	odds ratio	HPD^1^ 2.5%	HPD^1^ 97.5%
Intercept	1247677	−40.1		−40.3	−39.9
ln DIM	1247677	15.4		15.3	15.4
(ln DIM)∧2	1247677	−1.62		−1.62	−1.61
Parity 1	325621		*Reference*		
Parity 2	288951		1.056	1.006	1.109
Parity 3	223118		0.978	0.923	1.034
Parity 4	153753		0.948	0.888	1.010
Parity >4	256234		0.761	0.720	0.805
Year: 2002 or earlier	148578		*Reference*		
Year: 2003	86158		1.000	0.924	1.088
Year: 2004	147847		0.901	0.831	0.970
Year: 2005	216142		0.928	0.864	1.000
Year: 2006	313278		0.858	0.796	0.923
Year: 2007–8	335674		0.897	0.833	0.967
Season 1: Jan–Mar	332357		*Reference*		
Season 2: Apr–Jun	278139		0.897	0.857	0.938
Season 3: Jul–Sept	266050		0.736	0.701	0.775
Season 4: Oct–Dec	371131		0.997	0.957	1.040
Centred 305 d yield (×1000 kg)	1247677		0.917	0.906	0.928
No lameness 70-43 d before	1219868		*Reference*		
Lameness case 70-43 d before	27809		0.850	0.760	0.948
No lameness within 14 d	1207760		*Reference*		
Lameness case within 14 d	39917		0.760	0.686	0.839
No lameness 43–70 d after	1207155		*Reference*		
Lameness case 43–70 d after	40522		0.880	0.803	0.968
No lameness 71–100 d after	1203737		*Reference*		
Lameness case 71–100 d after	43940		0.861	0.787	0.947

1 HPD: interval of highest posterior density (so the range between HPD 2.5% and HPD 97.5% represents the 95% of the parameter space with highest posterior density).

### Probabilistic sensitivity analysis

#### Univariate analysis

**Figure 5:**
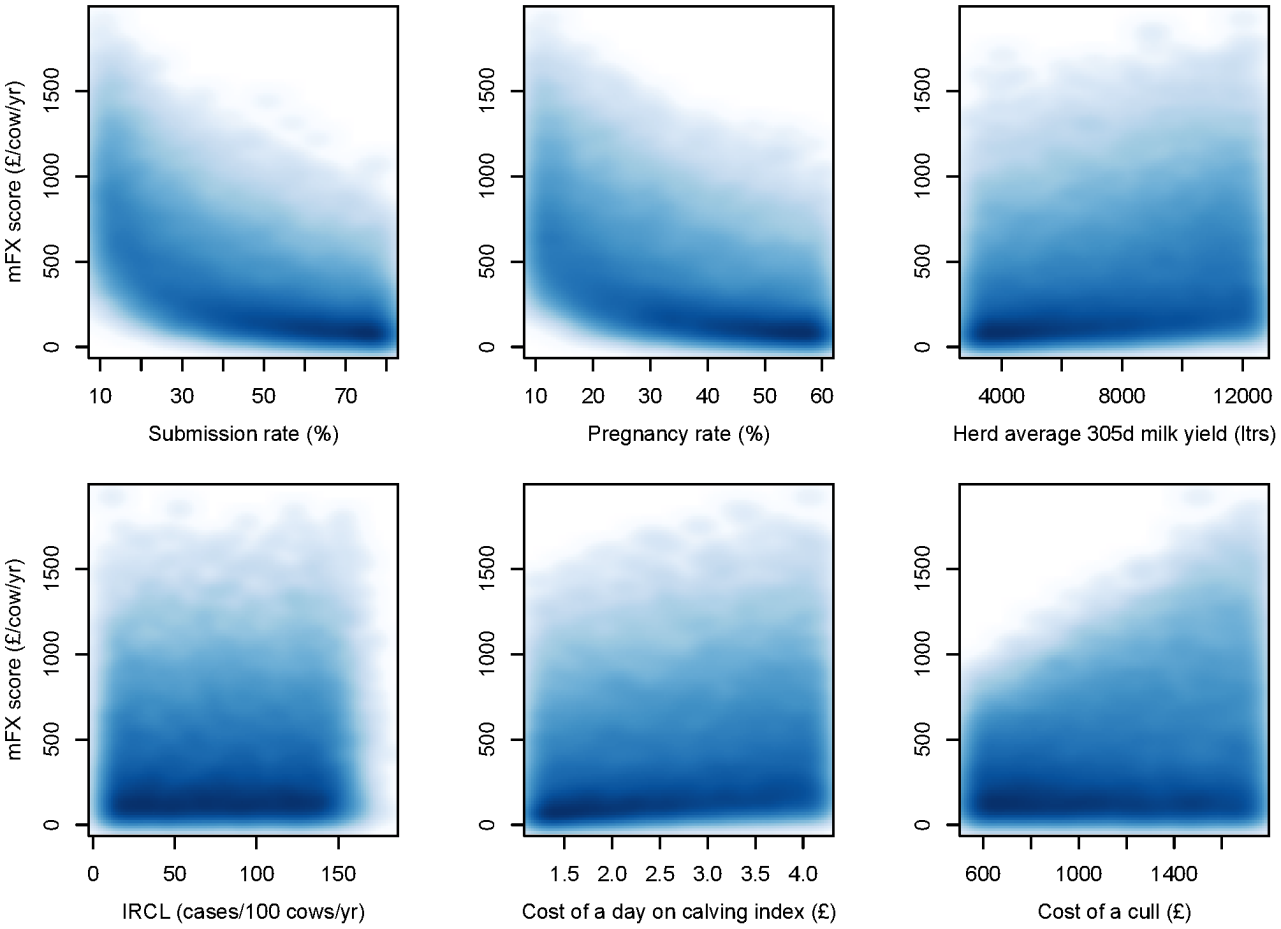
Associations between simulation inputs and overall herd-level reproductive performance. High density scatterplots showing the association between each simulated herd’s reproductive performance (represented by modified FERTEX score, mFX, y-axis) and selected simulation input variables. Darker colours indicate areas of higher point density, IRCL: Incidence rate of clinical lameness.

Univariate analysis of PSA results is presented using high-density scatterplots in Figure 5. These show that a herd’s “background” level of submission and pregnancy rate were the individual inputs with the strongest influence on overall herd fertility performance, with both being moderately strongly correlated with herd mFX score (Spearman rank correlation coefficient −0.65 for submission rate and −0.59 for pregnancy rate). The herd incidence rate of clinical lameness had no clear relationship with mFX score, with a Spearman rank correlation coefficient of 0.028 and the scatterplot showing a square appearance with no clear trend in the area of highest point density.

#### Multivariate analysis

Analysis of the simulation results in a multivariate framework allows visualisation of results from the discrete time survival model in a clinical context. Table 5 shows that the herd’s “background” level of submission and pregnancy rate explained the vast majority of the variation in herd mFX score, with 75% of overall variance explained by these two input parameters. It is important to remember that these inputs represent the marginal effect of between-herd variation in these aspects of fertility performance after the other model inputs have been accounted for (so, for example, a herd’s “background” pregnancy rate would reflect its insemination success rate after accounting for any effects of milk yield, age structure and level of lameness).

**Table 5:** Multiple regression derived partition of variance in modified FERTEX score across simulation input variables in a study evaluating associations between lameness and fertility in dairy herds.

Input parameter	Proportion of variance explained
Submission rate	41.4%
Pregnancy rate	34.2%
305-day adjusted lactation milk yield	8.9%
Cost per additional day on calving interval	5.7%
Cost per failure-to-conceive cull	2.0%
Incidence rate of clinical lameness	0.1%
Proportion of herd in lactation 1	0.0%

**Figure 6:**
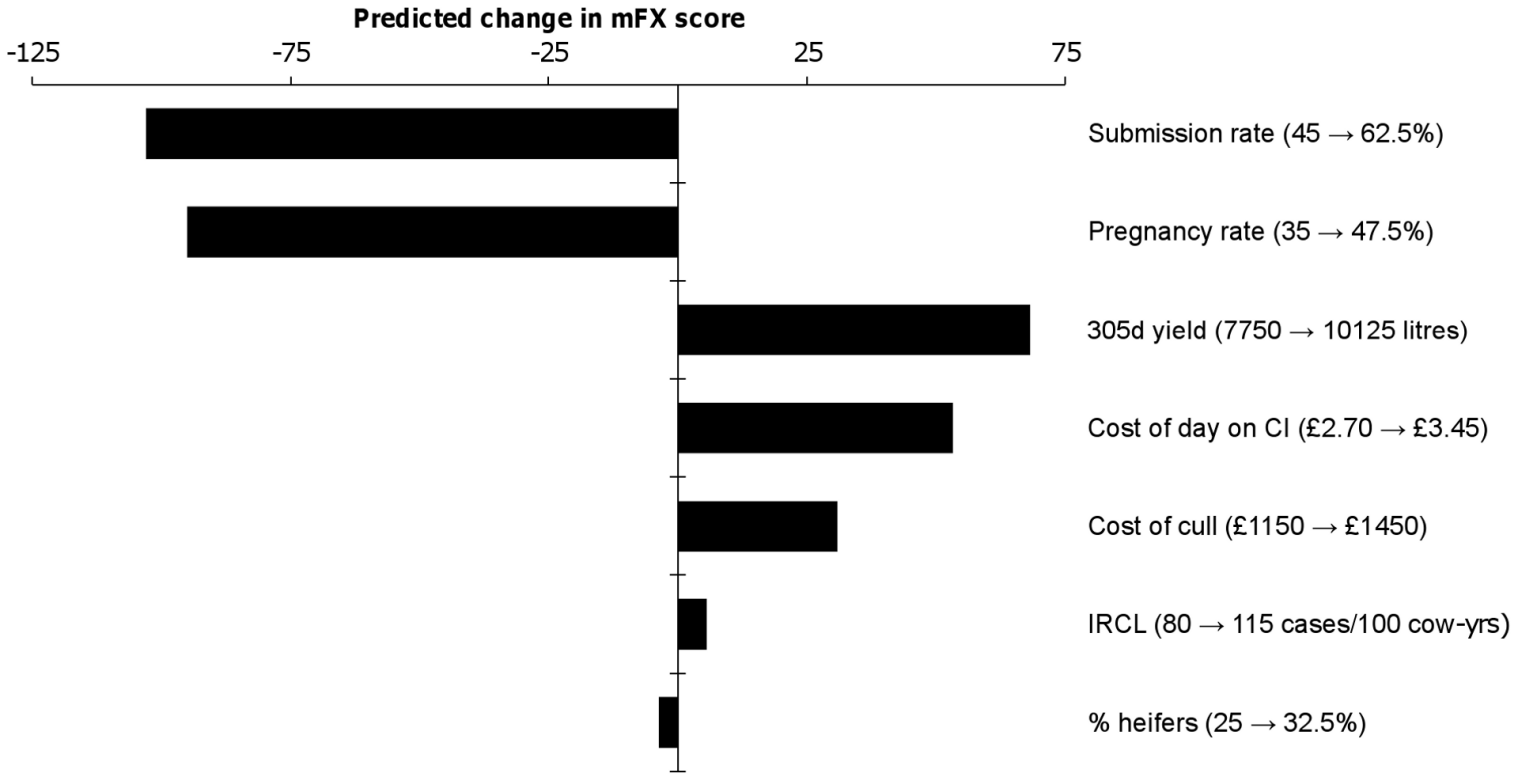
Predicted effect of an equivalent increase in each input parameter on overall reproductive performance. Tornado plot showing the predicted effect of increasing each input parameter in turn by a value representing 25% of the range of its input distribution from the median value, while the other input parameters are held at their population medians. The input parameters are listed on the right hand side of the graph, and the change in each input (from median to upper quartile) is given in parentheses. For example, the top bar shows that the predicted effect of moving from a submission rate of 45% (the median of the input distribution for this parameter) to 62.5% (the upper quartile of the input distribution) would be a decrease of just over £100/cow/year in the herd’s modified FERTEX (mFX) score.

Figure 6 shows the predicted change in herd mFX score which would result from a herd increasing each input parameter in turn from the middle of the range of the input distribution by 25% of the total range while the other inputs remain at the population median. For example, the top line on the plot shows that an increase in submission rate from the median value of the range of distribution for this input (45%) to the value representing the lower boundary of the upper quartile of the range (62.5%) would be expected to result in a decrease in mFX score (i.e. an improvement in overall reproductive performance) of around £100/cow/year. Increasing the herd’s incidence rate of lameness cases from 80 to 115 cases/100 cow-years would be expected to increase herd mFX score by just over £5/cow/year. Therefore, a reduction in lameness incidence of 35 cases/100 cow-years (which would represent a large improvement, and may require substantial financial and time investment from the farmer) would be expected to lead to the same degree of improvement in fertility as an increase in submission rate of less than 1% (a small change, which would be expected to require substantially less investment).

## Discussion

This study showed that relatively large associations between clinical lameness events and reproductive performance could be demonstrated at the level of a risk period within lactation (e.g. occurrence of a lameness case within 14 days of a risk period was associated with a 25% reduction in the risk of the cow becoming pregnant during the risk period, Figure 3). However, PSA revealed that a herd’s incidence rate of lameness was highly unlikely to make a significant contribution to its overall level of reproductive performance when other factors affecting fertility were also taken into account. As the simulation model was constructed to represent a herd with even all-year-round calving, it is possible that the results will be less applicable to block calving herds, where cows may have a limited timeframe in which to conceive. It is plausible that clinical lameness events may have an increased importance in the latter situation, as even a modest reduction in risk of pregnancy during the breeding period could increase the risk of a cow being culled.

There is substantial variation in the conclusions of existing work evaluating the association between lameness and reproductive performance. A variety of previous studies have found associations between decreased fertility and either clinical lameness events [2], [3], [25] and/or identification of lameness through visual gait assessment [26], [27]. In contrast, other studies have failed to reveal such an association [1], [18], [19]: notably there seems to be a tendency for studies involving larger numbers of herds to fail to identify significant associations. Many of the pre-existing papers in this area describe studies involving less than five herds (and most use a single herd); the notable exceptions to this are Loeffler et al. [18] (43 herds) and Sogstad et al. [19] (112 herds), neither of which found significant associations between lameness events and reproductive outcomes. It is biologically plausible that any effect of lameness on reproductive performance will vary between herds (for example, due to the variation in the predominant causes of lameness in each herd and variation in the effectiveness of management of lame cows). The current study used data from 39 herds, but from a much larger number of cows compared to previous work. The possibility of between-herd variability in the association between lameness events and fertility was explored here using herd-level random effects terms for the explanatory variables related to lameness. This revealed relatively little between-herd variability in effect within this group of herds. It is possible that somewhat different results would have been derived from building the statistical model using data from a different group of herds, but since the simulation model showed such a small potential for lameness to influence herd fertility, the difference in the statistical model results would have had to be extremely large in order to change the interpretation of the simulation results to any meaningful degree.

Some of the variation in previous published results may also be related to the way in which reproductive outcomes were measured: this study revealed significant associations between lameness events and the probability of pregnancy over a specific window of time relative to the lameness case, but when results were used to evaluate this within a PSA framework it transpired that lameness incidence rate was unlikely to influence overall herd reproductive performance. This means that previous studies focussing on particular categories or timings of lameness event and/or reproductive outcome may have been more likely to generate significant findings than those using broader categories or timeframes.

This study illustrates the usefulness of simulation-based techniques (such as PSA) to aid interpretation and contextualisation of model results. The approach we describe provides a potential route for researchers to facilitate better understanding of the results of their work and how they should be interpreted in a clinical context. This in turn can enhance research impact, and accelerate change in clinical practice. Although this example describes application of PSA to help interpret the results of a discrete time survival analysis, the technique would be equally applicable to other types of complex model, and to other analyses based on logistic regression. In logistic regression, the model coefficients themselves can be difficult to interpret. Classically the coefficients are exponentiated to produce odds ratios (as shown in Table 4), but odds ratios themselves can be misleading because humans intuitively tend to think in terms of risk or probability rather than odds (and these can be quite different, especially where the risk is close to 0.5). This topic has been extensively explored in the medical literature [28]–[30], where results of such analyses must be interpreted by clinicians, some of whom may have a limited understanding of statistical methods. It is possible to convert an odds ratio to a relative risk for more intuitive interpretation (as shown in Figure 3), but where decisions are to be made at population level these can also be difficult to interpret. For example, in this case the relative risks would have been hard to interpret without a method to incorporate the likely range of herd-level lameness incidence rates and the distribution of lameness events through lactation. Here, the results from the discrete time survival model alone (along with some of the pre-existing literature) may have encouraged clinicians to place too much emphasis on control of lameness to improve herd-level reproductive performance.

This study highlights the usefulness of simulation-based techniques such as PSA as an extension of statistical modelling to help illustrate model results in an intuitive way within a clinical veterinary context. In this example, while there are associations between lameness events and reproductive performance at specific time-points, it is unlikely that a herd’s incidence rate of lameness will have a substantial impact on herd fertility. This does not mean that lameness control is not important: lameness has significant impacts on both animal welfare and productivity [31]. Rather, our analysis suggests that herd lameness control is unlikely to lead to a significant improvement in overall reproductive performance in the majority of situations.
